# Atlanta Academy of Medicine

**Published:** 1874-08

**Authors:** A. W. Calhoun

**Affiliations:** Reporter


### Atlanta Academy of Medicine.

A. W. CALHOUN, M.D., Reporter.

Atlanta, June 1, 1874.

Dr. W. S. Armstrong, President, presiding.

Dr. Woodhull, U. S. A., read his essay on the Ligation of Arteries for the control of traumatic inflammation.

Dr. Calhoun asked for an expression of opinion on the part of the members of the Aademy in reference to a case about which he had been written to, with the view of operating, if it was now thought to be amenable to an operation. A young man (age not known) had, by an injury, suffered dislocation of the hip-joint, which had now existed ten or twelve weeks. The nature of the dislocation was not stated; the main inquiry being, could a dislocation of this joint, existing for such a time, be reduced?

Dr. Battey had a case, in 1860, which he reduced after six weeks standing, by simple manipulation. The limb was one-half inch elongated after reduction. Patient uses a stick and limps slightly, due probably to the elongation. Otherwise has done well up to the present time. Has had no other case, but thinks a reduction after eleven weeks not without precedent. Thought the case of Dr. Calhoun should be well considered before attempting a reduction, for the arteries and nerves in the region over which the head of the femur must play, before getting back into its proper position, would be very much endangered; also, the adhesions and other changes which may have taken place should be duly and carefully studied before deciding upon any operation. Dr. B.'s attempt at reduction in his own case was not sanctioned by his professional brethren, but he thought the trial justifiable, chiefly on account of the pecuniarily dependent condition of the patient, and would have made the effort had the prospect been even more gloomy. The man was quite poor and with a large family, which fact alone demanded the operation. Had he been a man of means, possibly nothing would have been attempted.

Dr. Armstrong agreed with Dr. Battey that all things should be thoroughly sifted before deciding. Thinks no positive answer should be given till the patient has been carefully examined.

Dr. Baird asked what difficulty was in the way of making the effort to reduce the dislocation, even though it did not succeed.

Dr. Battey replied: The adhesions may be so extensive and firm as to make it hazardous to break them up. The head of the femur might also be broken by the effort, particularly if the patient was an aged person. The danger of injuring the vessels and nerves by the movements of the head of the femur was not an unimportant thing. Thought manipulation the best method, but nothing short of a *determined* effort should be made, if anything at all is done.

Dr. Baird still insisted that in his opinion even a determined effort should be made in this case and not let the patient remain a cripple the balance of his life without a single chance having been given him.

Dr. Battey related an instance where an incautious attempt had been made to reduce a dislocated shoulder joint which only resulted in giving the patient much pain and after-trouble, and was the means of cutting him off from all hope of a successful effort in the future.

ATLANTA, June 8th, 1874.

Dr. Armstrong, President presiding.

Dr. Calhoun read the report of a case of trephining of the skull for the relief of epilepsy. Up to this time the operation has proved successful.

Dr. Battey was consulted by a seventeen year old young lady, on account of a very distressing disease. There existed a strange stare and an inability to close the lid of the left eye. In smiling the mouth was drawn around to the opposite or right side. Four years ago, while in perfect health, she went riding, and upon her return the family noticed the face drawn to one side. She knew nothing of it. Previously she had had some peculiar pains, but at this time none whatever. There was no drum in the left ear, and complete deafness. Menstruation had begun at the time of this sudden facial paralysis, four years ago, and since then it had been irregular, coming on with from four to seven weeks interval, and always accompanied with a good deal of pain. At present she has also leucorrhœa. Thinks the trouble about the face is evidently the result of paralysis of the portio dura, and that this is in some way connected with menstrual derangement. She presents a strumous appearance, and says that several members of her family have died of consumption. Examination of the chest does not reveal any important or well marked tubercular deposit. Paralysis of the portio dura from reflex action is usually of not so long duration as in this case. If it is uterine in its origin, thinks the case might be relieved by removing the uterine disorder, and by building up the general health by such remedies as cod-liver oil, fowler's solution, etc. The patient has already been under a tonic and electrical treatment for two or three years, but it has resulted in no good.

---

ATLANTA, June 15, 1874.

Dr. Taliaferro, Vice-President, presiding.

Dr. Kendrick exhibited the foot of a negro, amputated by Dr. Scott and himself, on account of a railroad accident. Pirogoff's operation was made. Negro doing well.

Dr. Taliaferro exhibited to the Academy a "new intra-uterine stem," devised by himself. It is made of watch spring, coated with gutta percha; is quite elastic and very light. It is easily and quickly made, by wrapping the steel spring with a strip of cloth, and frequently dipping it in a solution of gutta percha made with the sulphuret of carbon. It will be perceived that the instrument is flattened from before backwards. The instrument is retained in the uterus by passing a silver wire through one or both sides of the cervix, and also through the lower end of the stem. A compressed shot, upon one or both sides, as may be needed, secures the wire and stem perfectly in position. The presence of the instrument in the uterus and wire in the tissues cause not the least pain or inconvenience, and may be worn for an indefinite length of time, the patient going about at her usual occupation. By wrapping the instrument with copper wire, over small strips of zinc placed upon either side, a galvanic stem is made, that may be secured in the uterus and worn in the same way, and with perfect comfort and safety. He mentioned a case where the instrument had been used, and so far with excellent results.

Dr. Battey had seen the case referred to by Dr. Taliaferro, and thought the stem was working admirably well.

Dr. Woodhull had seen the same case later, and found her doing well. He thought it would be simpler and yet answer the same purpose, to pass the wire through one side only of the mouth of the uterus.

Dr. Woodhull asked if any one had had any experience in giving large doses of ipecac in dysentery in this region.

Dr. J. G. Westmoreland had heard of cases being treated by twenty-grain doses, and also thirty drops of laudanum, but with not very flattering results. He had thus treated one case himself, but had no opportunity of further watching it, as the excessive vomiting led the patient to apply elsewhere for relief.

Dr. Battey thinks the action might be good. He had often used ten-grain repeated doses, and found that the repetition sometimes dissipated the nausea brought on by the first dose.

Dr. J. G. Westmoreland had not observed such a tolerance of the remedy as mentioned by Dr. Battey; on the contrary, that each dose increased the nausea.

Dr. Cumming said ipecac was introduced as an anti-dysenteric remedy amongst the British troops in India. The results were excellent. He had used it years ago, and always found it to act beneficially. He tried to induce the surgeons in the Confederate army to use it, but they were too timid. When given in these large doses it acts as a purgative. Congestion of the liver in dysentery, in malarious regions, can be relieved by its use.

Dr. Battey asked if the stomach tolerated such doses.

Dr. Cumming replied: It is tolerated even to the extent of sixty or ninety grains. Give it as a powder, if possible; and always as little water as can be, for this oftentimes brings on the nausea.

Dr. Baird had seen iced-water injections recommended in dysentery. In a case treated by himself he had used carbolic acid injections, four or five drops at a time.

Dr. Woodhull, influenced by the reports of the British army, had tried the ipecac in several cases at the Barracks' Hospital with excellent results. He gives ten to fifteen drops of tinct. opii., and in half an hour afterwards twenty to thirty grains of the ipecac, in as little water as possible. He has his patients to lie quiet and drink no water. If the ipecac stays down the dysentery ceases. He had used it once in chronic dysentery with much benefit.

Dr. Cumming said: In very hot countries, when dysentery continues a few days, ulcers are sure to form; and though they may cure up, they are prone to re-open upon the slightest provocation. One of the objects of the ipecac treatment is to prevent the formation of ulcers.

Note.—Since the above was reported, Dr. Woodhull mentioned to the Reporter still another case of acute dysentery, treated in the last few days, by the use of ipecac, where the effect of the remedy was very marked. The bowels were acting frequently, and the patient was much reduced, having a pale, haggard and pinched expression of countenance. The laudanum was rejected by the stomach, which was very much irritated, when twenty-five grains of ipecac was at once given, quieting the stomach, and in a short time checking the bowels and changing the character of the actions to a healthy appearance. After a few hours, a second dose of thirty grains was administered, which was vomited, the patient having taken a large draught of water; but no further unpleasant effect followed. The patient recovered rapidly, having no other treatment.

---

Atlanta, June 22, 1874.

Dr. Armstrong, President, in the chair.

Dr. Battey presented the ovaries which he had removed from a patient on the previous Saturday by normal ovariotomy, Drs. Logan, Westmoreland, Kendrick and Griggs assisting. The case is one of long standing, chronic endometritis, which has resisted for ten or twelve years persistent efforts at cure. The case will be reported to the Academy in full at a future time. At present he only desired to call attention to the diseased state of the ovaries while they are yet fresh, and to the method of removal. The left ovary presents upon its surface two small cysts; there is likewise a similar cyst upon the right ovary. The three cysts are about the size of cherries, and contain a transparent liquid; one of them was ruptured in progress of the operation. The removal was effected with the patient in a semi-prone posture; the vagina opened with Sims' speculum; an opening was made into the utero-rectal pouch, in the median line, with scissors. The cervix uteri was now seized with a volselum, and drawn well down, whilst a finger was passed up into the pelvis, the left ovary seized and brought down in the vagina, and removed by *ecrasement*, without ligature being left on the pedicle. The right ovary was then treated in like manner. There was no disposition of the ovaries to prolapse into the wound; on the contrary, they required to be seized quite high up and brought down. There was a little oozing of bloody serum from the pedicles, but not enough to give any anxiety. The patient experienced subsequently a marked exemption from the severe pain which had attended his previous operations, and which he had attributed to the ligature left upon the pedicle. No suture was used in the vaginal wound. Up to the present the pulse has not exceeded eighty-four to the minute.

Dr. Taliaferro requested the information in detail in this case in reference to the endometritis. The diseased ovaries in this instance were most likely the result of disease of the uterus. Ordinarily endometritis is curable. Is ovariotomy to become a remedy for the cure of this disease? It is important to know *what*, and if *all* means have failed to bring about a cure. If all available means have really failed, then ovariotomy may be under some circumstances, justifiable. It must be remembered that what may be considered as exhausting all means by one physician, may be considered by another as far from being so.

Dr. Battey did not think the disease of the ovaries due to a disease of the uterus. Moreover, thought that long standing endometritis was not always so easily cured. Scanzoni admits that he never cured a single case, and is skeptical about reported cures. The patient has undergone every treatment for the disease. Derived most good from fuming nitric acid. The discharge is now slight and the parts look healthy, and the exquisite sensitiveness of the lining membrane of the uterus is gone. The great improvement in the uterus did not relieve the patient, and the ovariotomy was made to bring about permanent relief.

Dr. Taliaferro thought the close connection between the uterus and ovaries was clear evidence that diseased ovaries may come from a diseased uterus. Was of the opinion that the mild and often impotent remedies applied by Scanzoni was the cause of his not curing endometritis. Uterine therapeutics is in its infancy. The usual plans of treatment are far from thorough. He claims for the cloth tent a means of more thorough medication of the cavity of the uterus than has heretofore been attained, and that it is entirely safe.

Dr. Battey—As a rule, the wombs of women from whom diseased ovaries are removed are not diseased. Again, in diseases of the womb there is often no disease of the ovaries. Gaillard Thomas and Byford speak of long standing and severe cases, as if no cure was to be expected. Certainly no criticism can be brought to bear against Atthill's mode of treatment by nitric acid, for it is as complete and thorough as could be desired.

Dr. Taliaferro—An ulcer in the cavity of the uterus destroys the ciliary epithelium, and is replaced by a polymorphous variety. This is presumed to be the case when strong caustics are used, and this is an objection to Atthill's treatment. The Doctor holds firmly to the belief that no case of uncomplicated endometritis is incurable. Thinks the authorities will come to this opinion in a few years.

Dr. Rauschenberg—The main question is, whether the disease was originally in the uterus or ovaries. Would like to know the direct symptoms that prompt the removal of the ovaries, particularly when the uterus is diseased. Regards the uterus and ovaries as perfectly distinct. Disease of the one may exist, and not of the other.

Dr. Battey replied that the symptoms calling for ovariotomy were those calling for a change of life. This operation will bring on the change, and thinks the patient should have the benefit of it.

Dr. Rauschenberg—As a rule, menstruation and ovulation act in unison, but each can act separately. This is the opinion of the best European physiologists of the present day.

Dr. W. F. Westmoreland thought but little was known upon the subject of menstruation. He recently saw a case of normal ovariotomy in the hands of Dr. Thomas, of New York. The ovaries were removed last November, and the patient has menstruated regularly once a month since that time. Her general health has greatly improved, but not as much as she had hoped. He has always been of the opinion that ovulation was menstruation; if they are separate and distinct, he is at a loss to know how to come to any definite conclusion. He thinks Dr. Battey's operation will come into general use eventually.

Dr. Battey was asked to define "the change of life."

Dr. B. defines, when a woman loses the possible power of perpetuating the species, whether the loss be by nature or by art, she changes life. When a girl acquires the capability of impregnation, she has arrived at puberty, whether she stain her clothing or not; when a child-bearing woman loses all capability of impregnation, by atrophy or removal of the ovaries, she has *changed life*, whether she discharge blood from her uterus or not. Normal ovariotomy does not propose to arrest menstruation, in the popular use of the term menstruation, but only to effect the *change of life*, and thereby secure the altered nervous conditions which attend upon the climacteric. The existence or non-existence of an uterine epistaxis, whether regular or irregular, signifies nothing, so we have the nervous phenomena of the change of life, and the constitutional alterations incident thereto.

Academy adjourned until the first Monday in October next.

